# Stable Human Hepatoma Cell Lines for Efficient Regulated Expression of Nucleoside/Nucleotide Analog Resistant and Vaccine Escape Hepatitis B Virus Variants and Woolly Monkey Hepatitis B Virus

**DOI:** 10.1371/journal.pone.0145746

**Published:** 2015-12-23

**Authors:** Xin Cheng, Weiwei Guan, Shuo Sun, Baosheng Li, Haijun Li, Fubiao Kang, Jiwen Kang, Dongliang Yang, Michael Nassal, Dianxing Sun

**Affiliations:** 1 The Liver Disease Diagnosis and Treatment Center of PLA, Bethune International Peace Hospital, Shijiazhuang, PR China; 2 Troop 66220 of PLA, Xingtai of Hebei Province, PR China; 3 Department of Infectious Diseases, Union Hospital, Tongji Medical College, Huazhong University of Science and Technology, Wuhan, PR China; 4 Department of Internal Medicine II / Molecular Biology, University Hospital Freiburg, Freiburg, Germany; Indiana University, UNITED STATES

## Abstract

Hepatitis B virus (HBV) causes acute and chronic hepatitis B (CHB). Due to its error-prone replication via reverse transcription, HBV can rapidly evolve variants that escape vaccination and/or become resistant to CHB treatment with nucleoside/nucleotide analogs (NAs). This is particularly problematic for the first generation NAs lamivudine and adefovir. Though now superseded by more potent NAs, both are still widely used. Furthermore, resistance against the older NAs can contribute to cross-resistance against more advanced NAs. For lack of feasible HBV infection systems, the biology of such variants is not well understood. From the recent discovery of Na^+^-taurocholate cotransporting polypeptide (NTCP) as an HBV receptor new in vitro infection systems are emerging, yet access to the required large amounts of virions, in particular variants, remains a limiting factor. Stably HBV producing cell lines address both issues by allowing to study intracellular viral replication and as a permanent source of defined virions. Accordingly, we generated a panel of new tetracycline regulated TetOFF HepG2 hepatoma cell lines which produce six lamivudine and adefovir resistance-associated and two vaccine escape variants of HBV as well as the model virus woolly monkey HBV (WMHBV). The cell line-borne viruses reproduced the expected NA resistance profiles and all were equally sensitive against a non-NA drug. The new cell lines should be valuable to investigate under standardized conditions HBV resistance and cross-resistance. With titers of secreted virions reaching >3x10^7^ viral genome equivalents per ml they should also facilitate exploitation of the new in vitro infection systems.

## Introduction

Hepatitis B virus (HBV) is the type member of the *hepdnaviridae*, small hepatotropic DNA-containing viruses that replicate through reverse transcription [1,2]. The human virus causes acute and chronic hepatitis B (CHB), with an estimated 240 million chronic virus carriers worldwide [3] who are at greatly increased risk of developing end-stage liver disease [4]. Treatment of CHB was initially restricted to type-I interferon. The introduction in 1998 of lamivudine (LAM) marked nucleoside and nucleotide analogs (NAs) as an alternative. LAM was followed by adefovir (ADV), and more recently by entecavir (ETV), telbivudine (LdT) and tenofovir (TDF) [5,6]. ADV and TDF are acyclic nucleotide analogs, applied as more cell-permeable dipivoxil and disoproxil prodrugs. All these NAs target the viral polymerase and thus reduce reverse transcription of the viral pregenomic (pg) RNA into new relaxed-circular (RC) DNA genomes [2]. The error-prone replication mechanism enables HBV to quickly respond to selective pressure by evolving better adapted variants, as is evident from the emergence of drug-resistant virus variants under NA therapy. Upon 5 years of treatment, the cumulative annual incidences are ~70% for LAM and ~ 29% for ADV [6,7]. ETV and TDF suppress HBV replication more potently and thus are much less prone to resistance development; hence they are currently recommended as first line treatment for the management of CHB [4,8]. However, where successful, LAM with add-on ADV treatment is often continued, and both LAM and ADV are widely used in developing countries for economic reasons. Sustained virus elimination is rarely achieved with any of the NAs [9], implying a need for life-long treatment [10]. This aggravates the problem of cross-resistance [11], as illustrated by the massively enhanced emergence of ETV resistant variants in LAM experienced versus naive patients (51% vs. 1.2% after 5 years; [8]).

NA resistance is associated with specific mutations in the reverse transcriptase (RT) domain of the viral polymerase. Its 3D structure has not been solved yet, but the pertinent mutations occur within sequence motifs that are conserved in all reverse transcriptases and, where known, are part of catalytically important substructures such as the active site and the dNTP binding pocket. Modeling studies [12,13] and mutational analyses of the related duck HBV (DHBV) polymerase [14–16] suggest that this also holds for the corresponding HBV polymerase motifs. Primary resistance mutations may decrease viral fitness; this is often compensated for by subsequent secondary mutations.

Based on a unified RT domain numbering system [17], typical resistance mutations for LAM are rtM204I/V, rtL180M and rtA181T/V. ETV resistance requires at least 4 mutations yet two (rtM204I/V, rtL180M) are identical to those providing LAM resistance, lowering the barrier for ETV resistance. Mutations pertinent for ADV resistance include rtN236T and rtA181T/V [7].

Due to the overlapping polymerase and surface protein genes on the HBV genome, mutations in the RT domain can simultaneously affect the surface proteins (antiviral drug-associated S gene variants;[18]) and alter recognition by diagnostic and vaccination-induced antibodies [19,20]. Direct selection of S variants occurs in vaccinees and patients receiving anti-HBs immunoglobulin [21,22]. The mutations commonly reside in the major hydrophilic region of S (amino acids 99–169), particularly in the a-determinant (aa 124–147; [23]). The most frequent exchange is sG145R [24] yet many others are known, including sY100C [25] and sP120T [26]. Conversely, mutations in S can alter the polymerase and thus affect NA susceptibility.

Due to the primate-restricted host-range of HBV, phenotypic analyses of virus variants are mostly performed in surrogate systems. Besides primary human and *Tupaia* hepatocytes [27,28] an HBV infectable cell line, HepaRG, is available [29], yet as in primary hepatocytes there is no continuous virus propagation or net amplification. The recent identification of sodium taurocholate cotransporting polypeptide (NTCP) as an HBV entry receptor [30,31] enabled engineering of NTCP expressing hepatoma cell lines that become susceptible to HBV infection. However, infection rates of 50% or more of the cells currently require a very high multiplicity of infection (moi), usually indicated as viral genome equivalents (vge) per cell; typically used mois range from 500 to >10^4^ vge/cell [30–32]. Access to sufficient amounts of virus, especially defined variants, remains therefore a challenge.

HBV genome transduction with baculoviruses is rather efficient [33,34], but their construction is not trivial and baculo-HBV induces innate responses in HepaRG cells [35]. Transient transfection is simple but suffers from varying and hard to predict transfection efficiency. An alternative are stably HBV transfected human hepatoma cells such as the widely used HepG2.2.15 line [36] although these cells carry a complex multicopy HBV integrate and show altered proliferation and metastatic ability compared to parental HepG2 cells [37,38]. An advance came with regulatable systems, most notably TetOFF and TetON [39]. There, transcription of the HBV pgRNA is controlled by a tetracycline (Tet) responsive promoter (TRE), provided a Tet transactivator (tTA; a fusion protein of the Tet repressor and a transcription activation domain) is present in the same cell. In the TetOFF system, the tTA induces transcription only in the absence of tetracycline or its analog doxycycline (DOX).

A trade-off is the necessity to stably transfect the cells with expression cassettes for both the tTA and the TRE-controlled gene of interest. The simplest solution is a single-step double transfection, as used for the generation of cell lines encoding wild-type HBV, a single LAM resistant variant [40,41] or HBV defective in the synthesis of specific viral antigens [42], including in a murine hepatocyte-derived cell background [43]. However, besides a low frequency of the desired double transfectants, generation of any new cell line will lead to integration of the two expression cassettes into different genomic loci which may obscure virus-specific differences.

Using instead a sequential procedure we had previously established tTA expressing HepG2 and Huh7 TetOFF lines, and then used the best-performing clones as recipients for a TRE-controlled wild-type (wt) HBV genome [44]. The resulting cell lines HepG2.117 (which produces around ten times more HBV, infectious for primary tupaia hepatocytes, than HepG2.2.15 cells) and Huh7.93 have successfully been employed to address the impact of HBV replication on the cell and *vice versa*, including immunological [45,46] and metabolic issues [47] or, for the Huh7 line, as an HBV/HCV coinfection model [48].

Here we report on the generation of new cell lines, all in the same background of the tTA line HepG2.TA2-7, which produce six LAM and ADV resistance-associated and two vaccine escape variants of HBV and, in addition, woolly monkey HBV (WMHBV; [49]). WMHBV is distinct from all HBV isolates of humans and hominoid apes [50] and appears more infectious for *Tupaia* hepatocytes than HBV [51], providing a surrogate *in vivo* model in *Tupaia*-hepatocyte-xenotransplanted mice [52]. However, as woolly monkeys are endangered and WMHBV has been found in only few captive colonies, supplies of this virus are extremely limited. This has also hampered assessing the risk of WMHBV transmission into humans and exploring ways to prevent or treat accidental infection.

This panel of new cell lines should be useful for screening of novel antivirals with activity against LAM and ADV resistance-associated HBV variants, and for evaluating cross-resistance and drug susceptibility of major vaccine escape HBV variants. Furthermore, the cell lines secrete up to 3x10^7^ vge per ml culture supernatant as enveloped virions, suggesting them to aid in exploiting the emerging NTCP-based in vitro infection systems for better understanding of the biology of HBV variants as well as of WMHBV.

## Materials and Methods

This section contains condensed descriptions of pertinent experimental procedures. Additional detaills are provided as S1 Protocols.

### Plasmid constructs

All constructs were based on vector pTRE-HBVT [44] in which authentic wild-type HBV pgRNA of genotype D (Genbank accession no.: J02203) is transcribed from the TRE promoter; a hygromycin resistance gene serves as selection marker. RT mutations were introduced by mutagenic PCR, S gene mutations by replacing an ~0.6 kb restriction fragment covering nearly the complete S ORF by homologous fragments from a genotype A isolate carrying the sG145R mutation (accession no.: AF134134) and a genotype D isolate (accession no.: AF134141) carrying the sY100C plus P120T mutations [21]; both were kindly provided by W.F. Carman. The mutations are summarized in Table 1. For WMHBV, full-length viral DNA from serum of a WMHBV-infected woolly monkey was amplified and used to generate a vector analogous to pTRE-HBVT. The cloned WMHBV sequence differs at 7 positions from WMHBV2 (accession no.: AY226578.1).

**Table 1:** Amino acid and underlying nucleotide exchanges in the polymerase and S protein, and their effects on the overlapping ORF and encoded protein.

Amino acid exchange^a^	Codon change	Effect on overlapping ORF	Effect on overlappingly encoded protein
**rtV173L**	gTG > tTG	GAg > GAt	sE164N
**rtL180M**	cTG > aTG	TCc > TCa	sS181—*silent*
**rtA181T**	gCT > aCT	TGg > TGa	sW172***stop***
**rtM204I**	ATg > ATt	TgG > TtG	sW196L
**rtM204V**	aTG > gTG	ATa > ATg	sI195M
**rtN236T**	AaC > AcC	*no overlap*	*no overlap*
**sY100C**	TaT > TgT	CTa > CTg	rtL108—*silent*
**sP120T**	cCT > aCT	AcC > AaC	rtT128N
**sG145R**	gGA > aGA	CgG > CaG	rtR153Q

^a^Mutations in the polymerase are denoted by rt, those in the S protein by s. Positions of polymerase protein mutations are given in the RT domain numbering system [17].

### Cell culture, transfections, and clone selection

Cell culture, transfection and clone selection were performed as previously described [44], except that SYBR Green qPCR was used to pre-screen for HBV producing clones. Four qPCR positive clones per construct were further characterized by Southern blotting as described [53,54]. The clones showing the highest levels of HBV DNA production and strict DOX regulation were further expanded and used in the subsequent experiments.

### Native agarose gel electrophoresis (NAGE)

NAGE of equal aliquots of cytoplasmic lysates and blotting for immunological detection of viral capsids by anti-HBV core protein antibody [55] and of encapsidated DNA by molecular hybridization with a ^32^P labeled HBV DNA probe were performed as previously described [56,57].

### Drug susceptibility assays

Cells grown without DOX were seeded in 96-well plates at a density of 2×10^4^ cells/well. On the next day (day 1), cells were incubated in media containing serial dilutions of the desired drug; media were exchanged on day 3 and day 5. On day 7, cells were harvested and lysed using PBS buffer containing 0.3% NP40 detergent. After removal of the nuclei, HBV DNA was released from the cytoplasmic capsids by mixing 35 μl lysate with 65 μl BuccalAmp^TM^ (Epicentre, USA) and heat treatment as recommended by the supplier. DNA contents were determined by qPCR using a described Taqman procedure [58,59]. PCR primers corresponded to HBV positions 1603–1624 and 1661–1681 in the core start codon based numbering system [60]; the FAM/TAMRA labeled TaqMan probe covered HBV positions 1631–1656. All assays were performed in quadruplicate. Curve fitting by nonlinear regression and EC_50_ (effective concentration inhibiting virus replication by 50%) determinations were done using the log10 (inhibitor) vs. response equation implemented in Graphpad Prism V5 software. Fold resistance was calculated as the ratio of the EC_50_ for mutant vs. wild-type virus. Statistical significance was assessed by one-way ANOVA followed by Dunnet´s post test, with EC_50_ values for the wt-HBV line HepG2.117 line as reference. P-values <0.05 were considered significant.

### Quantification of enveloped viral particles released into the cell culture supernatant

Cells were passaged twice in DOX-free medium, and two days after the last splitting 100 μl supernatant each was treated with pronase and DNAse I to minimize contributions from DNA in extracellular non-enveloped nucleocapsids to the subsequent virion titer determinations [44]. After DNase inactivation by EDTA, DNAs in enveloped (i.e. protected) nucleocapsids were then extracted and quantitated using a commercial HBV DNA qPCR kit (Shanghai Kehua Bio-engineering, China) as recommended by the manufacturer. For comparison, analogous qPCR assays were also performed on culture supernatants not subjected to pronase/DNase treatment. All assays were performed in quadruplicate.

### Cytotoxicity assays

Cells were seeded in 96-well plates as for the drug susceptibility assays and maintained in the presence of increasing concentrations of the long-term stored ADV dipivoxil preparation (ADVdipi) used in the initial susceptibility assays, or free ADV (kindly provided by Gilead Sciences) used in the later assays. Dose-dependent cell viability was assessed in triplicate samples using the CCK-8 assay (Dojindo Laboratories, Japan) as recommended by the supplier. The cytotoxic concentration leading to death of 50% of the cells (CC_50_) was calculated analogously to the EC_50_ values using GraphPad Prism V5 software.

## Results

### Selection and characterization of well-regulated mutant HBV and WMHBV producing cell lines

**Fig 1:**
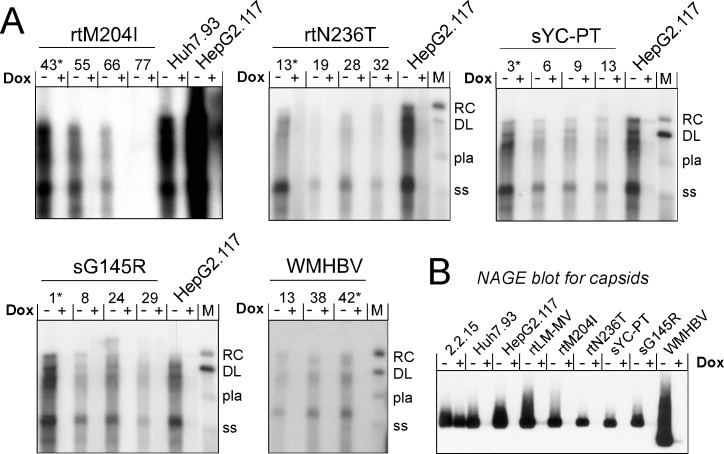
Generation of HepG2-based TetOFF cell lines producing variant HBVs and WMHBV. **(A) Selection of well-performing cell clones.** Clones preselected for the presence of HBV DNA were grown in the absence (-) or presence (+) of DOX. Intracellular capsid-associated viral DNA was analyzed by Southern blotting, using a ^32^P labeled HBV specific probe. The wt-HBV producing Huh7.93 and HepG2.117 lines [44] served as reference. M, marker DNAs consisting of mixture of a 3.2 kb linear HBV genome in double-stranded (DL) and heat-denatured single-stranded (ss) form, plus a 3.2 kb plasmid (pla) including about 500 bp HBV sequence. RC, relaxed circular DNA. Virus variants and individual clone numbers are given on the top, an asterisk indicates the clone used further; sYC-PT is the double-mutant sY100C-P120T. Data for the other cell lines are shown in S1 Fig **(B) Verification of DOX-regulation via capsid expression.** Equal aliquots of cytoplasmic lysates from the selected cell clones grown without or with DOX were separated by native agarose gel electrophoresis (NAGE). Capsids were detected using the anti-core protein monoclonal antibody mAb312 conjugated to peroxidase plus chemiluminescent substrate. The mAb´s epitope around aa 80 of the core protein is conserved in WMHBV core protein. Note the virtual absence of capsids in cells grown with DOX.

The nine derivatives of the original pTRE-HBVT vector [44] constructed encode variant HBV genomes with NA resistance-associated mutations (see Table 1) in the polymerase (rtM204I, rtL180M+M204V, rtV173L+L180M+M204V, rtN236T, rtA181T, and rtN236T+A181T), vaccine escape mutations in the S protein (sG145R, sY100C+P120T), and a replication-competent WMHBV genome. After transfection into the tTA containing HepG2.TA2-7 cells [44] and selection for hygromycin resistance, supernatants from up to 72 clones for each variant were prescreened, after DOX withdrawal, for HBV DNA by SYBR-Green qPCR. Four positive clones each were analyzed by Southern blotting to directly visualize replicative DNA intermediates. Several examples are shown in Fig 1A (for the remaining cell lines see S1 Fig). Nearly all candidate clones gave the expected DNA pattern in the absence but not in the presence of DOX, as desired. At least one clone each produced viral DNA in amounts between those observed for the wt-HBV lines Huh7.93 and HepG2.117 [44], and was chosen for further propagation (marked by an asterisk in Fig 1A).

Tight regulation of virus production by DOX was verified (Fig 1B) by native agarose gel electrophoresis (NAGE). In this assay, intact viral capsids migrate through the gel [55,57] and their positions and approximate amounts can be visualized by immunoblotting with capsid-specific antibodies. Because core protein is translated from the pgRNA, this provides a surrogate estimate for DOX-dependent regulation of pgRNA transcription. Expectedly, DOX addition did not detectably affect capsid levels in the non-regulated HepG2.2.15 cells but virtually abolished capsid formation in all of the new TetOFF cell lines. Icosahedral particle mobility in NAGE is principally determined by particle diameter and surface charge [61]; however, separation of the larger 240 subunit HBV capsids (triangulation number T = 4) from the smaller 180 subunit T = 3 HBV capsids requires agarose concentrations of >2% [62,63]. Hence the faster mobility of the WMHBV capsids observed in the 1% agarose gels used here suggests a higher negative surface charge compared to HBV capsids.

Finally, we assessed the amounts of enveloped virions secreted into the culture supernatants from the selected cell clones and, for comparison, from induced HepG2.117 cells. Transiently as well as stably HBV transfected cells can release non-enveloped capsids [42,64]. For the HepG2.117 and Huh7.93 cell lines such capsids accounted for at least half of the extracellular viral particles [44]. Prior to DNA isolation for qPCR, we therefore treated the secreted particles with pronase plus DNase [54] to degrade DNA in the protease-sensitive naked capsids whereas DNA in virions is protected by the lipid envelope. The protected DNA was then used as template for qPCR. For comparison, we performed analogous measurements with non-treated culture supernatants. As shown in Table 2, the pronase plus DNase treatment reduced the vge/ml values by about 2.5- to 5-fold for all cell lines encoding HBV with intact envelope protein genes. In contrast, for the two cell lines carrying HBV with the rtA181T mutation which causes premature translation termination in the overlapping S gene (sW172*; see Table 1), values after treatment were 1,600-fold (rtA181T) and 3,000-fold (rtA181T-N236T) reduced. Hence in accord with a previous study [65], the truncated envelope proteins can at most marginally support virion formation; also, we were unable to detect secreted HBsAg in the respective supernatants using a commercial chemiluminescent immunoassay (Abbott Architect). Based on the values after pronase plus DNase treatment, the supernatants from most of the new cell lines contained around 2–3 x10^7^ vge/ml, in a similar range as HepG2.117 cells (Table 2). Only for variants rtM204I and rtN236T were the yields lower (about ten-fold and four-fold, respectively). Together these data showed that all selected cell clones were able to produce substantial amounts of intracellular nucleocapsids and, where applicable, also of enveloped virions.

**Table 2:** Titers of HBV DNA in extracellular particles and LAM and ADV EC_50_ values for cell line-encoded wt-HBV and HBV variants.

Cell line	Extracellular HBV DNA^a^		LAM		ADV	
	(-) pronase / DNase	(+) pronase / DNase	EC_50_ ± SD^b^ [μM]	Signi-ficancevs. wt^c^	EC_50_ ± SD^b^ [μM]	Signi-ficancevs. wt^c^
**HepG2.117 (wt-HBV)**	1.6 ±0.8 x10^8^	3.1 ±1.1 x10^7^	0.34 ± 0.16	n.a.	0.39 ± 0.12	n.a.
**rtM204I**	8.4 ±2.3 x10^6^	3.4 ±1.6 x10^6^	33.04 ± 4.94	***	0.66 ± 0.20	ns
**rtL180M-M204V**	6.6 ±2.5 x10^7^	2.2 ±1.1 x10^7^	35.94 ± 5.76	***	0.59 ± 0.36	ns
**rtV173L-L180M-M204V**	6.0 ±2.3 x10^7^	2.4 ±0.8 x10^7^	22.58 ± 8.08	***	0.65 ± 0.35	ns
**rtA181T** ^**d**^	4.7 ±1.6 x10^6^	***2*.*9 ±1*.*0 x10*** ^***3***^	8.51 ± 3.65	*	14.59 ± 7.60	*
**rtN236T**	3.2 ±5.5 x10^7^	8.1 ±2.5 x10^6^	0.28 ± 0.22	ns	23.57 ± 9.92	***
**rtA181T-N236T** ^**d**^	2.4 ±0.9 x10^6^	***0*.*8 ±0*.*4 x10*** ^***3***^	10.12 ± 2.93	**	26.35 ± 11.7	***
**sG145R**	1.5 ±0.5 x10^8^	2.9 ±1.2 x10^7^	0.70 ± 0.49	ns	0.57 ± 0.12	ns
**sY100C-P120T**	9.9 ±3.3 x10^7^	3.0 ±1.0 x10^7^	0.55 ± 0.27	ns	0.55 ± 0.29	ns

^a^ In vge/ml culture supernatant ±SD by qPCR of DNA without (-) or with (+) prior pronase/DNase treatment.

^b^ EC_50_ values [μM] calculated using the log10(inhibitor) vs. response equation implemented in GraphPad Prism 5, based on quadruplicate determinations of intracellular HBV DNA by qPCR.

^c^ Significance of differences between mean EC_50_ values for variant vs. wt-HBV cell line as calculated by one-way ANOVA and Dunnet´s post test (GraphPad Prism 5); n.a., not applicable; ns, not significant

***, p ≤ 0.001

**, p ≤ 0.01

*, p ≤ 0.05. Scatter blots showing the four individual LAM and ADV EC_50_ determinations for each cell line from which mean values and significance were calculated are provided in S2 Fig

^d^ Cell lines encoding HBV with the rtA181T/sW172* mutation did not produce detectable HBsAg; the drastic drop in extracellular HBV DNA after pronase/DNase treatment indicates that nearly all extracellular DNA was associated with non-enveloped capsids.

### Susceptibility of stably expressed HBV variants to LAM treatment

**Fig 2:**
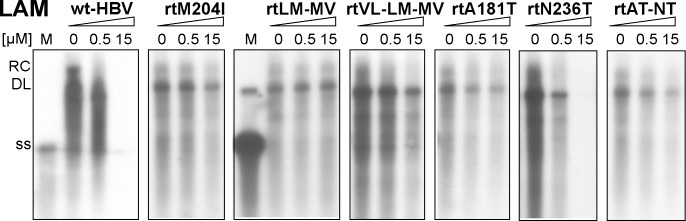
Semiquantitative assessment of LAM resistance by Southern blotting. Cells producing the indicated viruses grown in the absence of DOX were treated, or not, with 0.5 μM or 15 μM LAM. Intracellular viral DNAs were analyzed as in Fig 1A, except that a heat-denatured 3.2 kb HBV restriction fragment served as marker for ss DNA (strong signal) and DL DNA (weak signal). Note the nearly complete loss of signal at 15 μM drug for the wt-HBV and rtN236T lines, but not the cell lines encoding viruses with established LAM resistance-associated mutations. EC_50_ values for all cell lines are provided in Table 2.

As a first test for NA susceptibility of the cell line encoded viruses we used Southern blotting to visualize viral DNA from intracellular nucleocapsids after the cells had been kept for 7 days in the presence of 0, 0.5 or 15 μM LAM. As shown in Fig 2, wt-HBV was highly sensitive, with almost no viral replicative intermediates detectable at the high LAM concentration. The S gene variant and WMHBV cell lines gave comparable results (not shown). In contrast, much less pronounced reductions at high LAM concentration were observed for the variants bearing the LAM resistance-associated mutations rtM204I, rtL180M+M204V, rtV173L+L180M+M204V. Some resistance was also evident for variants rtA181T and rtN236T+A181T, but not rtN236T alone, all in accord with published data.

For a more quantitative evaluation we next determined the levels of intracellular HBV vge, using a wider concentration range of LAM (0.1 to 25 or up to 125 μM). The mean EC_50_ (effective concentration causing a 50% reduction) values ± standard deviation (SD: n = 4) are summarized in Table 2; scatter blots showing the underlying individual results are provided in S2 Fig Consistent with the Southern blotting data (Fig 2) the LAM resistance-associated mutations increased the EC_50_ from about 0.35 μM for wt-HBV to 23 to 35 μM (about 65- to 100-fold resistance) for the mutants comprising the rtM204I exchange, and to 8 to 10 μM (about 25-fold resistance) for those involving the rtA181T exchange. The differences in mean EC_50_ for these variants versus wt-HBV were all statistically significant. All other variants, including rtN236T, showed wt-HBV-like EC_50_s below 1 μM.

### Seemingly low ADV resistance of HBV variants with ADV resistance-associated RT mutations upon treatment with long-term stored ADV dipivoxil

ADV resistance was addressed analogously, initially using ADV dilutions prepared from a stock solution of ADV dipivoxil that had been kept at -20°C for four years (termed ADVdipi for distinction from the later experiments). Southern blotting after incubation at 0.5 and 15 μM of this preparation suggested a 50% signal reduction at around 1 μM of ADVdipi for wt-HBV and all variants bearing non-ADV-resistance mutations, i.e. in an expected range. Unexpectedly, however, no sign of resistance was seen for the ADV resistance-associated mutants; also EC_50_ determinations by qPCR showed on very slight increases (1.3- to 1.5-fold; data not shown). A potential mix-up of cell clones was excluded by sequencing the relevant part in HBV-specific PCR products from the rtN236T line. Furthermore, no substantial ADVdipi resistance was observed upon transient transfection of an rtN236T HBV construct into HepG2 cells (data not shown).

**Fig 3:**
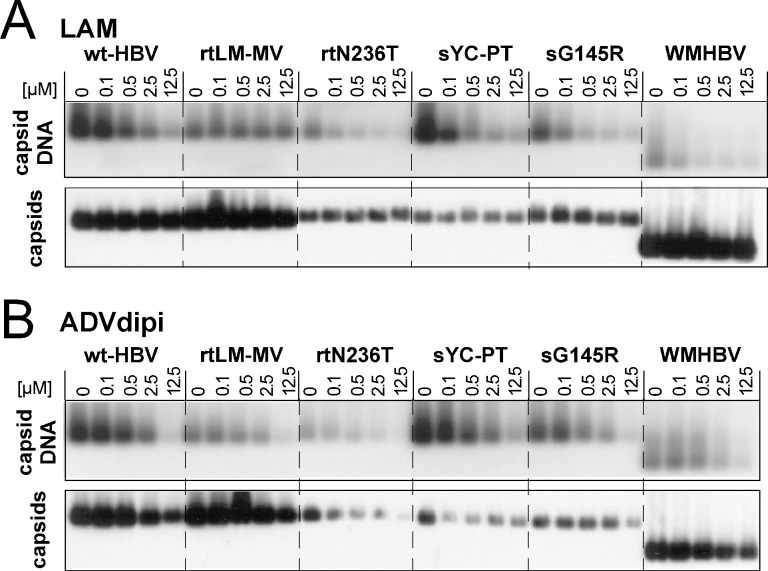
Simultaneous detection of capsid-associated DNA and capsids by NAGE blotting suggests cytotoxicity of long-term stored ADV dipivoxil. Cell lines for the indicated HBV variants and WMHBV were treated with LAM or a long-term stored ADV dipivoxil (ADVdipi) preparation. Equal aliquots from cytoplasmic lysates were separated on 1% agarose gels and blotted onto Nylon membranes (for capsid DNA) or PVDF membranes (for capsids; as in Fig 1B). Capsid-associated DNAs were detected using the same ^32^P labeled DNA probe as for Southern blotting. **(A) LAM.** LAM reduced the DNA signals in all samples except those with LAM-resistance associated variants but had no impact on capsid levels, as expected. **(B) ADVdipi.** ADVdipi reduced both DNA and capsid levels, consistent with a general cytotoxic effect. A semiquantitative evaluation is provided in S3 Fig

Suspecting a cytotoxic effect of the ADVdipi preparation we employed the NAGE assay to directly compare the levels of capsids and capsid-associated viral DNA. Blocking reverse transcription, NAs are expected to reduce viral DNA in the capsid but not to substantially affect capsid levels. Several of the cell lines were therefore treated with either ADVdipi, or with LAM as a reference (Fig 3). LAM caused a dose-dependent reduction in viral DNA for all variants except rtLMMV but only a marginal decrease in capsid levels at 12.5 μM concentration, as expected. In contrast, ADVdipi dose-dependently reduced viral DNA yet also capsids for all variants, including rtN236T (Fig 3B). Semiquantitative evaluation by densitometric scanning of the immunoblot bands confirmed this result, with a 50% reduction occurring in all cases between 2.5 and 12.5 μM ADVdipi; a graphical representation of the different impacts of LAM vs. ADVdipi is shown in S3 Fig Lastly, direct cytotoxicity testing using the CCK-8 assay on the rtA181T line revealed a CC_50_ of 7.15± 1.83 μM for the ADVdipi preparation.

A likely explanation for the cytotoxicity of the ADVdipi preparation, namely decomposition of the dipivoxil moietey with formation of formaldehyde as toxic principle, is provided in the Discussion section.

### Pronounced resistance of HBV variants with ADV resistance-associated RT mutations upon treatment with pure ADV

**Fig 4:**
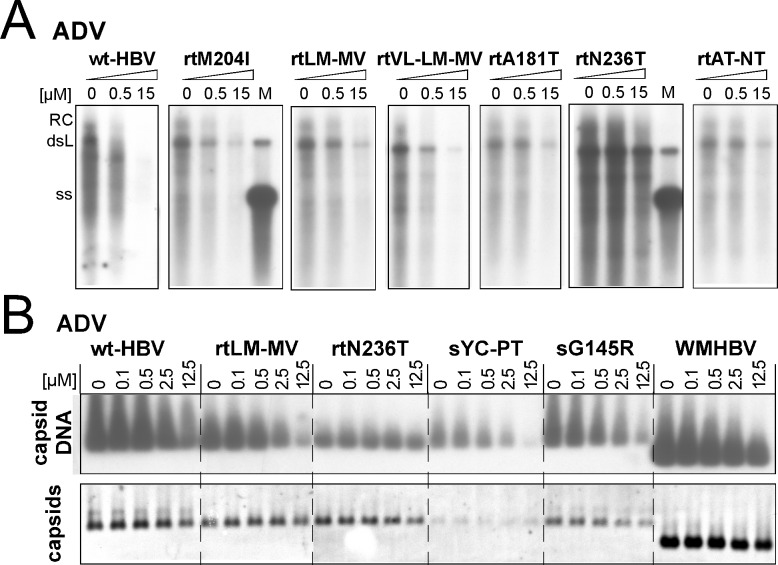
Pronounced resistance of cell line-borne HBVs carrying established ADV resistance-associated mutations against pure ADV. The indicated cell lines were treated with pure ADV at the indicated concentrations. Drug susceptibility was assessed by Southern blotting (A) and NAGE blotting (B). Note the marked loss of capsid DNA signals but at most slight decrease in capsid signals at the highest ADV concentration for all cell lines except the rtN236T line. EC_50_ values for all cell lines are provided in Table 2.

Next we repeated the experiments using a fresh preparation of free ADV. Cytotoxicity determinations showed mean CC_50_ values of 103.5±4.9 μM for the HepG2-117 line, 96.4±3.9 μM for the rtA181T line, 127.0±4.2 μM for the rtN236T line, and 116.6±4.0 μM for the rtA181T+N236T line, and thus well above the EC_50_ of ADV expected for wt-HBV. Southern blotting (Fig 4A) revealed clear to strong reductions of the DNA signals from the low (0.5 μM) to the high (15 μM) ADV dose for wt-HBV and the LAM resistant variants; in contrast, signals were much less reduced for those bearing ADV resistance-associated mutations, e.g. rtN236T. In the NAGE assay (Fig 4B) this preparation of ADV resulted in a strong, dose-dependent reduction of capsid-associated viral DNA for all tested cell lines except rtN236T, whereas at most minor reductions in capsid levels were observed at the highest ADV concentration. In particular, resistance of the rtN236T variant was now clearly evident (compare the rtN236T panels for ADVdipi versus ADV in Figs 3B and 4B). In addition, these results confirmed semiquantitatively the sensitivity of the S gene variants as well as of WMHBV to both LAM and ADV.

Quantitative assessment by qPCR confirmed a significant variant-specific ADV resistance (Table 2). Compared to HepG2.117 cells (EC_50_ 0.39±0.12 μM), the rtN236T and rtA181T+N236T lines were much less sensitive (EC_50_ 23.57±9.92 μM and 25.36±11.70 μM, respectively; p<0.01); a lower though significant (p<0.05) resistance was observed for the rtA181T line (EC_50_ 14.59±7.55 μM). In contrast, viruses carrying LAM resistance-associated mutations or mutations in S showed similar sensitivity as wt-HBV (EC_50_s <1 μM; see Table 2 for an overview). Thus the new HBV cell lines are suitable to address the viruses´ sensitivity to known drugs.

### Similar sensitivity of cell line-borne variant HBVs and WMHBV towards a non-NA compound targeting the capsid

A potential application of our variant HBV cell lines is to evaluate the activity of antivirals whose mechanism of action differs from that of NAs. As an example we here used the capsid-targeting compound Bay41-4109 [66], a heteroaryldihydro-pyrimidine (HAP) derivative. HAP compounds affect capsid assembly by inducing formation of irregular multimers or, at higher concentration, by disassembly of preformed capsids [67]. As hepadnaviral reverse transcription occurs inside capsids, capsid disintegration also prevents accumulation of viral DNA.

**Fig 5:**
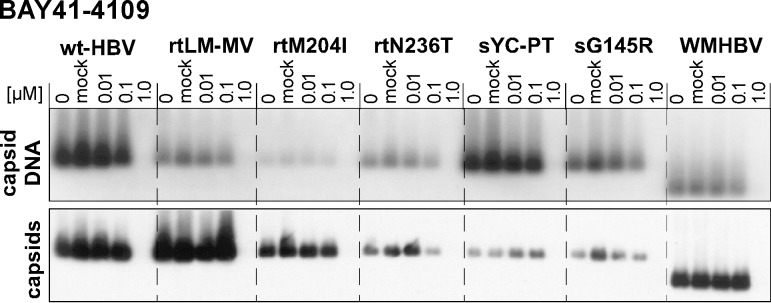
Cell line-borne HBVs with NA resistance-associated and S mutations as well as WMHBV are similarly sensitive to BAY41-4109 as wt-HBV. The indicated cell lines were treated with BAY41-4109 at the indicated concentrations; mock refers to treatment with 0.1% (v/v) DMSO vehicle alone. Equal aliquots from cytoplasmic lysates were analyzed for capsid DNA and capsids as in Fig 3. Note the strong decline in both capsids and capsid DNAs between 0.1 to 1.0 μM, as expected from the drug´s mechanism-of-action.

Based on previous data for HepG2.117 cells [44] we treated representative members of the new cell lines with Bay41-4109 at concentrations from 0.01 to 1 μM. As the solvent DMSO substantially increases HBV replication in HepG2.117 cells at concentrations above 0.5% [44] we kept the DMSO concentration below 0.1% and also used 0.1% DMSO as empty vehicle control. As shown in Fig 5, all tested cell line-encoded viruses, including NA resistant and S-gene variants as well as WMHBV responded with an almost complete loss of capsids, and consequently capsid-associated DNA, at 1.0 μM drug. DMSO alone (Fig 4, lane mock) had no detectable impact. While detection of more subtle differences in drug susceptibility would require testing of additional drug concentrations, in particular in the 0.1–1.0 μM range, the data including the steep response curves are fully in line with our previous results in the wild-type HBV cell line HepG2.117 [44] as well as the reported EC_50_ value of 0.2 μM in HepG2.2.15 cells [68].

Thus NAGE provides a simple assay to semiquantitatively assess sensitivity of HBV variants against different types of drugs, with the simultaneous detection of both capsids and encapsidated DNA providing additional mechanistic information.

## Discussion

The widespread clinical use of NAs for CHB treatment has generated large reservoirs of drug-resistant virus variants, particularly against LAM and ADV. Resistance against these older drugs can severely impact the efficacy of newer ones [7]. Furthermore, drug-induced polymerase mutations and vaccine escape mutations in S can mutually affect each other [7,18,19]. Better understanding the biology of these variant viruses is therefore mandatory for knowledge-based management of the disease as well as for the development of further improved drugs.

Stably transfected HBV producing cell lines have become an important tool in HBV and anti-HBV drug research. The uniform production of virus in essentially all cells of a culture, and the one or few copies of chromosomally integrated viral DNA versus microgram amounts of plasmid used in transient transfection greatly facilitate standardization. Regulatable HBV production permits, in addition, kinetic analyses in synchronized cultures, and the adjustment of HBV production per cell by varying the DOX concentration [44]. Not the least, our previously established HepG2.TA2-7 tTA cell line allowed us to introduce eight other, variant HBV genomes as well as WMHBV into the very same cell background.

### Well replicating and well controlled variant HBV and WMHBV cell clones

As expected from transfecting the new pTRE vectors into the pre-established HepG2.TA2-7, ≥10% of the hygromycin resistant clones contained intact HBV expression cassettes leading to viral replication. In at least one clone per construct, the levels of viral replicative DNA intermediates were between those in the wild-type HBV lines Huh7.93 and HepG2.117 (Fig 1A; S1 Fig), previously determined to be (without enhancement by DMSO) around 25 copies and 140 copies per cell [44]. All selected clones displayed strict DOX regulatability (Fig 1A and 1B) and the levels of HBV replication were easily sufficient for quantitative assessment of drug sensitivity by qPCR yet also for Southern blotting. This also held for the WMHBV cell line. Furthermore, all clones carrying virus genomes with an uninterrupted S ORF secreted very substantial amounts of enveloped virions into the culture supernatants. Hence the TetOFF HepG2 cell lines as such can be used to study intracellular virus replication and its inhibition, and the high levels of defined virions they release should be valuable for future infection experiments.

### Suitability of the new cell lines for drug testing

Usefulness of the new cell lines for drug testing was demonstrated by the distinct susceptibility profiles of the cell line-encoded viruses to LAM and ADV (Table 2), and to the non-NA inhibitor BAY 41–4109. Regarding LAM, the qPCR data were fully corroborated by Southern blotting (Fig 2) and NAGE analysis (Fig 3A). The LAM EC_50_ value of ~0.3 μM for the wt-HBV producing HepG2.117 cells is congruent with our previous estimates [44] and with data obtained by others [58] in HepG2 cells stably transfected with non-regulated HBV constructs (0.11 μM). The cell line-borne viruses with classical LAM resistance-associated mutations displayed strongly increased tolerance (up to 100-fold) towards LAM (Table 2, and Figs 2 and 3A). Also variants containing the rtA181T exchange (rtA181T and rtA181T+N236T) showed significantly higher LAM EC_50_ values than wt-HBV (Table 2), as reported [58]; however, we did not observe LAM resistance for the rtN236T mutation alone. We also note that another study [69] reported an even enhanced LAM susceptibility of the closely related A181V variant, and differing levels of LAM resistance (1.7-fold to 7.7-fold) have been observed with clonal HBV isolates from a single patient which all contained this mutation [70]. Numerous factors are likely responsible for these differences, including genetic differences outside the pertinent resistance-related regions, or the specific cell line used; for instance, LAM EC_50_ values may differ up to thousand-fold between Huh7 [71] and HepG2 [72] cells (2.5 μM vs. 0.03 μM). Additional variability may come from the use of different expression systems and assay formats. Our stable cell lines could substantially reduce such variability.

Regarding ADV, the initial susceptibility assays were obscured by cytotoxicity of the ADV dipivoxil preparation used (see below). However, with pure ADV Southern blotting and NAGE assays (Fig 4) as well as the qPCR results (Table 2) clearly confirmed a reduced susceptibility of the viruses carrying known ADV resistance-associated mutations. Hence the viruses encoded by our new cell lines faithfully reproduce the pertinent NA resistance features. The new cell lines should therefore become useful tools to address cross-resistance against other NAs. We have not explicitly measured EC_50_ values of LAM and ADV for the WMHBV cell line; however, semiquantitatively (Figs 3A and 4B), the monkey virus was similarly sensitive to both drugs as wt-HBV.

Treatment with BAY 41–4109 as an example for a non-NA antiviral [66] revealed a similar response for all viruses, including WMHBV, with a steep decline in capsid and capsid-borne viral DNA between 0.1 and 1 μM concentration (Fig 5). Given the compound targets core protein, this was not surprising for the HBV variants which all contain the identical core protein sequence. Notably, though, the WMHBV core protein differs from that of HBV at 19 aa positions, including 13 in the assembly domain; hence these residues are not crucial for drug binding, even if more precise EC_50_ determinations than were done here would reveal a slightly reduced susceptibility to the drug. Testing new HBV antivirals against WMHBV may thus be useful for mechanism-of-action studies. Altogether, these data underlined the suitability of the new cell lines for testing the efficacy of non-NA drugs in the presence of NA resistance.

### Caution is advised when using long-term stored solutions of ADV dipivoxil for drug susceptibility testing

Cytotoxicity of the initially used ADVdipi preparation suspected from concomitant declines in both capsid DNA and capsid levels (Fig 3B, S3 Fig) was directly confirmed by direct cytotoxicity testing, with a CC_50_ of around 7 μM. It has previously been noted that ADV dipivoxil, but not ADV itself, was cytotoxic for mouse macrophages and splenocytes at 15 μM and 30 μM concentration [73]. This has been ascribed to formaldehyde as one of the decomposition products of the (bis)pivaloyloxymethyl (dipivoxil) ester moiety. A similar mechanism is likely for our ADVdipi preparation. As the rate of decomposition will be affected by numerous parameters, it seems advisable to subject ADV dipivoxil stock solutions, especially upon longer storage, to direct cytotoxicity testing. In contrast, the CC_50_ of pure ADV (about 100 μM) was sufficiently high to allow unambiguous detection of ADV resistance for the pertinent virus variants (Fig 4; Table 2).

### NAGE as a complementary assay to qPCR for drug resistance assessment

The accuracy of the NAGE assay does not match that of qPCR for quantitative virus determination. Its strengths, however, are simplicity, requiring only small amounts of cytoplasmic lysate which can directly be loaded on the agarose gel, and the extra information provided on capsid levels as a second parameter (Fig 3, Fig 4). Beyond detecting the unexpected impact of ADVdipi on capsid levels this is exemplified by the results obtained with BAY 41–4109 which would immediately have suggested the capsid as target of the drug. Hence in combination with our new cell lines the assay appears not only useful for assessing novel NA inhibitors but also other classes of inhibitors. Virologically, the NAGE data confirmed that all mutant HBVs as well as WMHBV remained highly sensitive to at least one NA and to BAY 41–4109.

### Anticipated value of the new cell lines as a permanent source of defined viruses for infection studies

Human NTCP (over)expression renders HepG2 cells susceptible to HBV infection [30,31], promising to make in vitro infection assays much more widely accessible [74]. The studies published thus far, however, commonly indicate that infection rates in the range of 50% or more of the cells require a several hundred- if not ten-thousand-fold excess of virus particles (measured as vge) over cells [30,32]. Hence a single experiment in one well of a 24-well plate with 5x 10^5^ cells may consume in the order of 10^10^ vge [30]; for high-throughput approaches the demand will be proportionally higher. Patient sera may contain sufficient levels of virus but are not routinely available and contain heterogeneous virus populations. Hence stable cell lines provide a more feasible alternative, as illustrated by the use of wild-type HBV derived from the HepAD38 cell line in most current studies [30–32]. This line produces around ten-fold more virus than HepG2.2.15 cells [41], very similar to our HepG2.117 line [44]. Hence the production of nearly equal levels of secreted virions by various of our new variant cell lines (Table 2) suggests their usefulness for future infection studies. Notably, yields may further be increased by extending the time period for virion accumulation, or by inclusion of 1% DMSO in the growth medium which boosts virion production by HepG2.117 cells about five-fold [44].

The S variant cell lines and the virions they secrete should allow to assess how the surface protein mutations affect secretion of subviral HBsAg particles and virions [75] and the interaction with entry-relevant host factors including NTCP and glucosaminoglycans [76], thus helping to improve diagnostics and antibody-based immunoprophylaxis. The new WMHBV cell line provides permanent access to this rare virus, facilitating basic research on its biology as well as on clinical aspects such as transmissibility to humans. Specifically, our data already suggest that LAM and ADV would provide an effective prophylaxis upon accidental exposure to WMHBV-positive blood or blood-products, e.g. of zookeepers in contact with WMHBV-positive woolly monkeys.

## Supporting Information

S1 Fig Selection of TetOFF HepG2 cell clones producing additional HBV variants with NA resistance associated mutations. (PDF)

S2 Fig Scatter blot of individual cell line-specific EC_50_ determinations. (PDF)

S3 Fig Reduced capsid levels upon treatment with ADVdipi but not LAM suggest cytotoxicity. (PDF)

S1 Protocols (PDF)
