# Supplementary material for: Stable Human Hepatoma Cell Lines for Efficient Regulated Expression of Nucleoside/Nucleotide Analog Resistant and Vaccine Escape Hepatitis B Virus Variants and Woolly Monkey Hepatitis B Virus
Source: PLoS One. 2015 Dec 23;10(12):e0145746. doi: 10.1371/journal.pone.0145746 (PMC4689378; doi:10.1371/journal.pone.0145746)
Supplement: S1 Fig — (PDF) [file pone.0145746.s001.pdf]

S1 Fig.

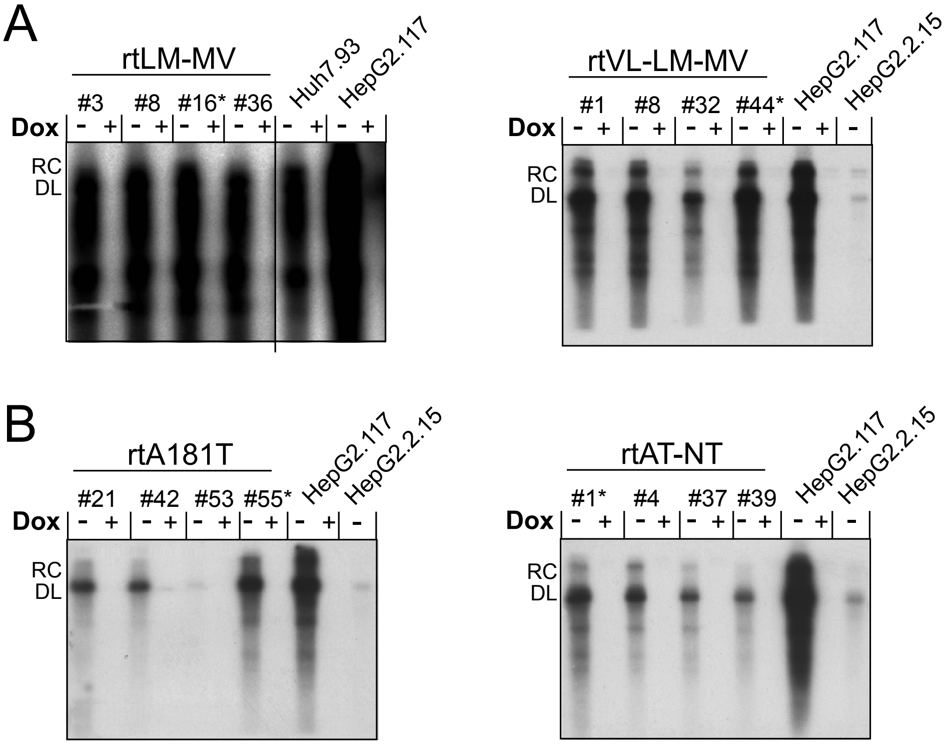

S1 Fig. Selection of TetOFF HepG2 cell clones producing additional HBV variants with NA resistance associated mutations. Intracellular capsid-associated DNAs from four cell clones per construct grown in the absence (-) or presence (+) of DOX were analyzed by Southern blotting as in Fig. 1 A using a ^{32}P labeled HBV DNA probe. DNA from the wild-type HBV producing cell lines HepG2.117, Huh7.93 and HepG2.2.15 served as reference. RC and DL denote the positions of relaxed circular and double-stranded linear forms. **(A) LAM resistance-associated variants.** rtLM-MV, rtL180M-M204V; rtVL-LM-MV, rtV173L-L180M-M204V. **(B) ADV resistance-associated variants.** rtAT-NT, rtA181T-N236T. Clones marked by an asterisk were further propagated and used in the subsequent experiments.
