# Supplementary material for: Stable Human Hepatoma Cell Lines for Efficient Regulated Expression of Nucleoside/Nucleotide Analog Resistant and Vaccine Escape Hepatitis B Virus Variants and Woolly Monkey Hepatitis B Virus
Source: PLoS One. 2015 Dec 23;10(12):e0145746. doi: 10.1371/journal.pone.0145746 (PMC4689378; doi:10.1371/journal.pone.0145746)
Supplement: S2 Fig — (PDF) [file pone.0145746.s002.pdf]

S2 Fig.

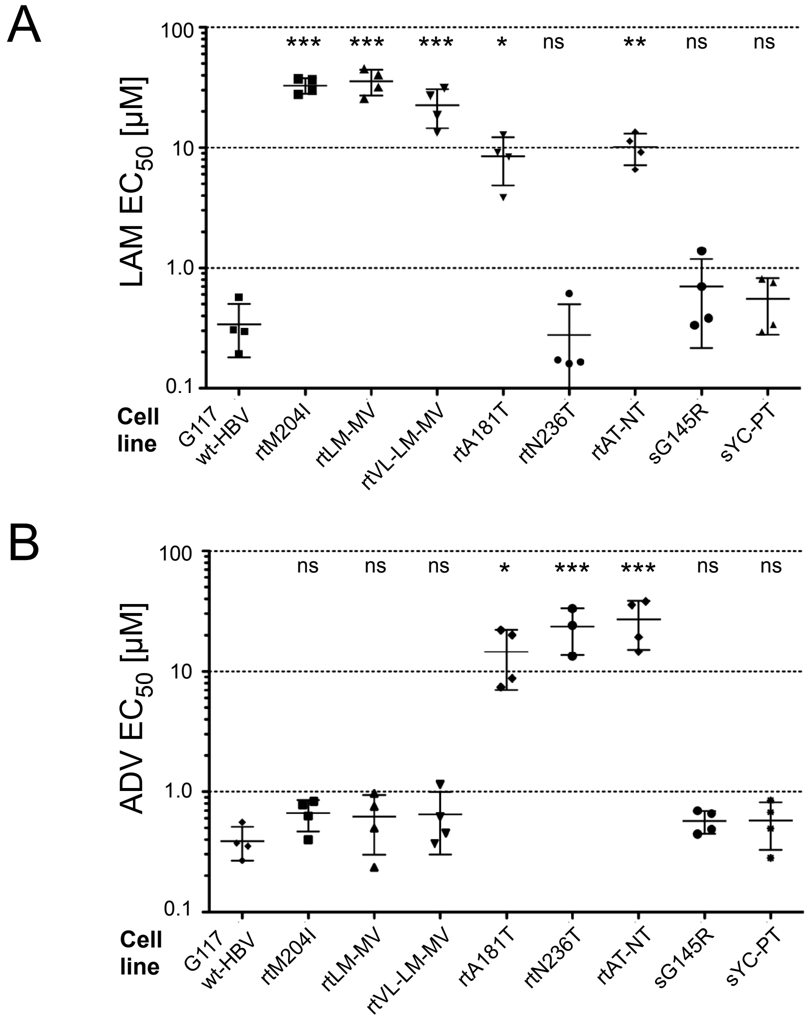

S2 Fig. Scatter blot of individual cell line-specific EC_{50} determinations. (A) Lamivudine (LAM). (B) Adefovir (ADV). Data points represent individual EC_{50}s as measured by four independent qPCR determinations of viral genome equivalents in the indicated cell lines upon treatment with LAM or ADV, and data evaluation by the log (inhibitor) vs. response function implemented in Graph Pad Prism. Horizontal bars show the mean value, vertical bars SD. Significance of differences in the mean EC_{50} values for the mutant HBV cell lines versus wild-type (wt) HBV in the HepG2.117 line was assessed by One-way ANOVA, using Dunnett´s multiple comparison post-test (Graph Pad Prism). Significance levels are: ***, $p<0.001$; **, $p<0.01$; *, $p<0.05$; ns, not significant.
