# Supplementary material for: Stable Human Hepatoma Cell Lines for Efficient Regulated Expression of Nucleoside/Nucleotide Analog Resistant and Vaccine Escape Hepatitis B Virus Variants and Woolly Monkey Hepatitis B Virus
Source: PLoS One. 2015 Dec 23;10(12):e0145746. doi: 10.1371/journal.pone.0145746 (PMC4689378; doi:10.1371/journal.pone.0145746)
Supplement: S3 Fig — (PDF) [file pone.0145746.s003.pdf]

S3 Fig.

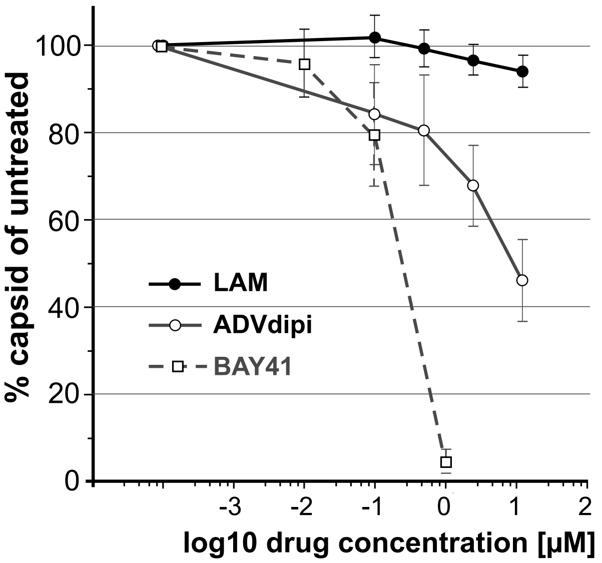

S3 Fig. Reduced capsid levels upon treatment with ADVdipi but not LAM suggest cytotoxicity. Capsid signals from the immunoblots in Fig. 3A (LAM), Fig. 3B (ADVdipi) and Fig. 5 (BAY 41-4109) were quantified by densitometry. For each cell line, the signal remaining at a given drug concentration was normalized to that in the untreated control which was set at 100%; the curve shown represents the mean reduction in capsids over all cell lines tested. Error bars represent standard deviations. LAM treatment induced only a minor, non-significant reduction even at the highest concentration. In contrast, the reduction by 12.5 µM ADVdipi to about 50% of the capsid levels of the untreated controls was statistically significant, suggesting a cytotoxic effect. BAY41-4109 induced a massive loss of capsids between 0.1 µM to 1.0 µM concentration which exactly paralleled the loss of capsid-associated DNA, consistent with the drug´s mechanism-of-action.
