# Supplementary material for: Stable Human Hepatoma Cell Lines for Efficient Regulated Expression of Nucleoside/Nucleotide Analog Resistant and Vaccine Escape Hepatitis B Virus Variants and Woolly Monkey Hepatitis B Virus
Source: PLoS One. 2015 Dec 23;10(12):e0145746. doi: 10.1371/journal.pone.0145746 (PMC4689378; doi:10.1371/journal.pone.0145746)
Supplement: S1 Protocols — (PDF) [file pone.0145746.s004.pdf]

S1 Protocols

Plasmid constructs. The parental vector pTRE-HBVT (1), based on plasmid pTRE2hyg (Clontech), contains a 1.05x HBV genome of genotype D (Genbank accession no.: J02203), subtype ayw (2), in which authentic pgRNA is transcribed from the TRE promoter. All mutations in the RT domain were introduced by standard mutagenic PCR. Presence of the desired mutations was confirmed by DNA sequencing.

The specific RT domain mutations (corresponding nucleotide exchanges are given in parentheses; altered nucleotides in lower case) were rtV173L (gTG>tTG), rtL180M (cTG>aTG), and rtM204I (ATg>ATt) for LAM; rtA181T (gCT>aCT) and rtN236T (AaC>AcC) for ADV. The corresponding six HBV variants were M204I; L180M+M204V; V173L+L180M+M204V; A181T; N236T; and A181T+N236T. Amino acid numbering is based on the consensus nomenclature where the first residue of the highly conserved motif EDWGPCDEHG is assigned position 1 (3). For the vaccine escape variants, nearly the complete S gene (positions 1437-2117 in the numbering system (4) in which the A residue of the core ORF ATG marks position 1) from the Avr II site (position 1460) to the Bsr GI site (position 2050) was replaced by corresponding fragments from a genotype A isolate carrying the sG145R mutation (accession no. AF134134) and a genotype D isolate (accession no. AF134141) carrying the sY100C (TaT>TgT) plus P120T (cCT>aCT) mutations (5); both were kindly provided by W.F. Carman. For the sG145R construct, the original nucleotide sequence specifying S amino acids D144 and R145 was GAt aGA, causing a premature stop in the overlapping polymerase ORF (underlined); to rescue polymerase production, the D144 codon was changed to GAc, as present in the parental pTRE-HBVT vector; this exchange causes an R488Q (R153Q in the RT nomenclature) in the polymerase. Resequencing revealed the presence of two additional amino acid exchanges in S (GtT>GgT, sV190G; cCA>gCA sP203A) compared to the Genbank sequence; these were not further modified. In total, the S protein in the construct differs at 17 positions from that in the wild-type HBV

sequence present in the HepG2.117 line, and these cause 16 exchanges in the polymerase between positions 388 to 546 (53 to 201 in the RT nomenclature).

WMHBV. Full-length viral DNA from serum of a WMHBV-infected woolly monkey was PCR-amplified using primers WMHBV fl(+)3 CTCACCATGCATCTTTTTCACCTCTGCCTGAT (WMHBV-2 1810-1841) and WMHBVfl(-)2 CATGGTGAGGCAGTTCCCACCAAT (complement to WMHBV-2 1795-1818). Restriction sites for Kpn I and Sac I encoded upstream of the virus specific sequences in the primers were used to clone the PCR products. Replication competence of several clones was assessed after recloning as 1.05x genomes into a CMV promoter driven expression plasmid, pCWM-9/3088, homologous to the HBV vector pCH-9/3091 (6). The sequence of the best performing clone (7) differs at 7 positions from that of WMHBV-2 (A1662G; A2322G; A2564G; A2582G; T2584G; A2607G; A2622G). Plasmid pTRE-WMHBV was assembled analogously to pTRE-HBVT using conventional DNA cloning procedures.

Cell culture, transfections, and clone selection. The HepG2.TA2-7 cell line (1) contains a stably integrated gene for the tTA2 version (8) of tTA, providing for high level and strict Dox-controlled gene expression. Cells were cultured in a fully humidified atmosphere containing 5% CO_2, 95% air at 37℃ in high glucose Dulbecco's minimal essential medium (Thermo Scientific) supplemented with 10% heat-inactivated fetal bovine serum (Thermo Scientific), 1% (v/v) non-essential amino acids (Invitrogen), 2 mM L-glutamine, 100 µg/mL of streptomycin, and 100 U/mL of penicillin G, buffered with sodium bicarbonate. As a precaution against inadvertent selection of altered cell clones, cells were passaged no longer than 3 months and then were replaced by low passage cells from frozen stocks.

For transfection with the pTRE-HBV vectors, exponentially growing HepG2.TA2-7 cells were seeded in 6-well plates at 2 × 10^5 cells per well, grown for 24 h, then transfected using FuGene™ 6 reagent as recommended by the manufacture (Roche). Cells were trypsinized after 24h and seeded into selective media containing 200 µg/mL of G418 (Sigma-Aldrich), 150 µg/mL of hygromycin and 2 µg/mL doxycycline. Media were renewed every third day.

After 3 weeks, 48-72 resistant colonies for each variant were picked into 24-well plates and cultured in selective medium without doxycycline to induce HBV expression. Similar numbers of cells were seeded into fresh 24-well plates for quantification of extracellular HBV DNA by quantitative PCR (qPCR) with SYBR green reagents (see below) or dot blot hybridization. The four clones from each variant cell line showing the highest levels of HBV DNA were further passaged for 20 generations (about 4 months) in hygromycin-free medium without loss of regulatable HBV expression.

Quantitative PCR (qPCR) screening for well expressing cell clones. Viral DNA was extracted from 200 µL of cell culture supernatant using the QIAamp™ DNA Mini Kit (Qiagen, Hilden, Germany) according to the manufacturer's instructions. DNA was eluted into 100 µL nuclease-free water, of which 5 µl was added to a 25 µl PCR reaction containing a commercial SYBR-Green reaction mix (Qiagen). HBV primers were 20922 (positions 1258–1290; numbering system of Pasek et al. (4) in which the A of the core initiation codon is assigned position 1) and -20988 (complementary to positions 1718–1740) (7). Thermal cycling was performed in a SLAN™ Real-Time PCR system (Hongshi, Shanghai, China). Reaction conditions were: 95℃ for 15 minutes followed by 40 cycles of 94℃ for 15 seconds, 55℃ for 30 seconds and 72℃ for 30 seconds. Each run included a dilution series of known amounts of a 3.2kb Eco RI restriction fragment covering the entire HBV genome for calibration, and supernatant derived from the parental HBV-free HepG2.TA2-7 cell line as negative control.

Detection of replicative HBV DNAs by Southern blotting. Southern blotting was performed as previously described (7, 9). In brief, cytoplasmic extracts were obtained by resuspending the cells in lysis buffer (10 mM Tris-HCl [pH 8.0], 1 mM EDTA [pH 8.0], 150 mM NaCl, 0.2% NP-40) and removing nuclei and cellular debris by centrifugation for 2 min at 13,000 rpm at 4°C. To the supernatants, Mg^{2+} acetate (final conc. 6 mM), DNase I (final conc. 200 µg/mL), micrococcal nuclease (final conc. 20 U/mL) , and RNase A (final conc. 1 mg/mL) were added, and the mixture was incubated at 37°C for 45 min to digest

nonencapsidated DNA. After EDTA (final conc. 10 mM) was added, capsid-protected DNA was released by proteinase K (final conc. 800 μg/mL) and SDS (final conc. 0.5%) for 3~5 hours at 45℃. After phenol/chloroform extraction, HBV DNA was precipitated using 0.1 volumes of 10 M ammonium acetate, GlycoBlue (Ambion, final conc. 60 μg/mL) and 1 volume isopropanol, and washed by 70% ethanol. After gel electrophoresis, HBV specific DNAs were detected using a ^{32}P-labeled probe obtained by random priming (NEBlot^{TM} Kit) on a 3.2 kb Eco RI fragment containing a complete linear HBV genome.

Native agarose gel electrophoresis (NAGE). NAGE was performed with aliquots from cytoplasmic extracts as previously described (7, 9) except the extracts were supplemented with a protease inhibitor cocktail (Roche). For semiquantitative comparisons, the aliquots were adjusted for equal total protein content, as confirmed by Western blotting against β-actin. Samples were electrophoresed through 1% agarose gels. For detection of HBV capsids, gels were blotted to a PVDF membrane, followed by incubation with the HBV core protein specific monoclonal antibody mAb 312 (10) conjugated to peroxidase. Bands were visualized using a chemiluminescent substrate (ECL+, GE Healthcare) and X-ray film.

For detection of encapsidated viral DNA, gels were blotted on a positively charged nylon membrane (Roche) by capillary transfer in TNE buffer (10 mM Tris-HCl [pH7.5], 150 mM NaCl, and 1 mM EDTA). Capsids were broken up by soaking the membrane for 15 s in 0.2 M NaOH, 150 mM NaCl. After neutralization in 0.2 M Tris-HCl [pH 7.5], 1.5 M NaCl , the membrane was fixed by UV-cross-linking (1.5 J cm^{-2}) and HBV-specific nucleic acids were detected using the same ^{32}P labeled probe as used for Southern blotting.

Antivirals susceptibility assay. Cell lines producing wild-type and mutant HBV were assayed in parallel. Cells were seeded in 96-well plates at a density of ~2×10^4 cells/well and allowed to attach overnight. On the following day (day 1), the cells were incubated with fresh media containing serial dilutions (usually 0.1, 0.5, 2.5, and 12.5 μM final conc.) of the desired drug; media were exchanged on day 3 and day 5. As we had previously demonstrated a strong enhancement of HBV replication in HepG2.117 cells by DMSO at concentrations of

0.5% and higher (1), for the BAY41-4109 experiments the final DMSO concentration was kept below 0.1%. On day 7, cells were washed twice with PBS and lysed in PBS with 0.3% NP40. The lysates were spun at 500 g for 5 min to pellet nuclei. HBV DNA was released from the cytoplasmic cores by mixing 35 µl of each lysate with 65 µl BuccalAmp™ (Epicentre) and heat treatment as recommended by the manufacturer. DNA contents were determined by qPCR using a previously described Taqman procedure (11). PCR primers were 5' CAA CCT CCA ATC ACT CAC CAA C 3' (HBV positions 1603-1624) and 5' ATA TGA TAA AAC GCC GCA GAC AC 3' (complementary to positions 1661-1681); the FAM/TAMRA labeled TaqMan probe had the sequence 5' TCC TCC AAT TTG TCC TGG TTA TCG CT 3' (HBV positions 1631-1656) (12). PCR reactions were performed in a 20 µl volume using 10 µl of 2x TaqMan Master Mix (Applied Biosystems), 200 nM labeled probe, 300 nM HBV forward primer, 300 nM reverse primer, and 2 µl of extracted DNA template. All determinations were performed in quadruplicate. Curve fitting by nonlinear regression and EC50 (effective concentration of drug inhibiting virus replication by 50%) determinations were done using the log (inhibitor) vs. response equation implemented in Graphpad Prism 5 software. Fold resistance was calculated as the ratio of the EC_{50} for mutant vs. wild-type virus. Statistical significance was assessed by one-way ANOVA followed by Dunnet´s post test as implemented in Graphpad Prism 5 software, with EC_{50} values for the HepG2.117 (wild-type HBV) line as reference. P-values of $p<0.05$ were considered significant.

Supplementary references:

1. **Sun D, Nassal M.** 2006. Stable HepG2- and Huh7-based human hepatoma cell lines for efficient regulated expression of infectious hepatitis B virus. J Hepatol. **45**:636-645.
2. **Galibert F, Mandart E, Fitoussi F, Tiollais P, Charnay P.** 1979. Nucleotide sequence of the hepatitis B virus genome (subtype ayw) cloned in E. coli. Nature. **281**:646-650.
3. **Stuyver LJ, Locarnini SA, Lok A, Richman DD, Carman WF, Dienstag JL, Schinazi RF.** 2001. Nomenclature for antiviral-resistant human hepatitis B virus mutations in the polymerase region. Hepatology. **33**:751-757.
4. **Pasek M, Goto T, Gilbert W, Zink B, Schaller H, MacKay P, Leadbetter G, Murray K.** 1979. Hepatitis B virus genes and their expression in E. coli. Nature. **282**:575-579.

5. **Ireland JH, O'Donnell B, Basuni AA, Kean JD, Wallace LA, Lau GK, Carman WF.** 2000. Reactivity of 13 in vitro expressed hepatitis B surface antigen variants in 7 commercial diagnostic assays. Hepatology. **31**:1176-1182.

6. **Nassal M, Rieger A.** 1996. A bulged region of the hepatitis B virus RNA encapsidation signal contains the replication origin for discontinuous first-strand DNA synthesis. J Virol. **70**:2764-2773.

7. **Sun D, Rösler C, Kidd-Ljunggren K, Nassal M.** 2010. Quantitative assessment of the antiviral potencies of 21 shRNA vectors targeting conserved, including structured, hepatitis B virus sites. J Hepatol. **52**:817-826.

8. **Baron U, Gossen M, Bujard H.** 1997. Tetracycline-controlled transcription in eukaryotes: novel transactivators with graded transactivation potential. Nucleic Acids Res. **25**:2723-2729.

9. **Ren S, Nassal M.** 2001. Hepatitis B virus (HBV) virion and covalently closed circular DNA formation in primary tupaia hepatocytes and human hepatoma cell lines upon HBV genome transduction with replication-defective adenovirus vectors. J Virol. **75**:1104-1116.

10. **Sällberg M, Ruden U, Magnius LO, Harthus HP, Noah M, Wahren B.** 1991. Characterisation of a linear binding site for a monoclonal antibody to hepatitis B core antigen. J Med Virol. **33**:248-252.

11. **Weinberger KM, Wiedenmann E, Bohm S, Jilg W.** 2000. Sensitive and accurate quantitation of hepatitis B virus DNA using a kinetic fluorescence detection system (TaqMan PCR). J Virol Methods. **85**:75-82.

12. **Qi X, Xiong S, Yang H, Miller M, Delaney WE 4th.** 2007. In vitro susceptibility of adefovir-associated hepatitis B virus polymerase mutations to other antiviral agents. Antivir Ther. **12**:355-362.
